# The association of loneliness and social isolation with healthcare utilization in Denmark

**DOI:** 10.1093/eurpub/ckac130.186

**Published:** 2022-10-25

**Authors:** J Christensen, SS Pedersen, CM Andersen, P Qualter, R Lund, M Lasgaard

**Affiliations:** 1 Department of Psychology, University of Southern Denmark, Odense, Denmark; 2 Public Health and Health Services Research, Defactum, Central Denmark Region, Aarhus, Denmark; 3 Department of Cardiology, Odense University Hospital, Odense, Denmark; 4 Manchester Institute of Education, University of Manchester, Manchester, UK; 5 Department of Public Health, University of Copenhagen, Copenhagen, Denmark; 6 Center for Healthy Aging, University of Copenhagen, Copenhagen, Denmark; 7 Steno Diabetes Center Odense, Odense University Hospital, Odense, Denmark

## Abstract

**Objectives:**

The present prospective cohort study investigated the association of loneliness and social isolation (SI) with healthcare utilization (HCU) in the general population over time.

**Methods:**

Data from the 2013 Danish “How are you?’ survey (n = 29,472) were combined with individual-level register data from the National Danish Patient Registry and the Danish National Health Service Registry over a 6-year follow-up period (2013-2018). Negative binomial regression analyses were performed while adjusting for baseline demographics and chronic disease.

**Results:**

Loneliness measured at baseline was significantly associated with more GP contacts (incident-rate ratio (IRR) = 1.03, 95% confidence interval (CI) [1.02, 1.04]), more emergency treatments (IRR = 1.06, 95% CI [1.03, 1.10]), more emergency admissions (IRR = 1.06, 95% CI [1.03, 1.06]), and hospital admission days (IRR=1.05, 95% CI [1.00, 1.11]) across the 6-year follow-up period. No significant associations were found between social isolation and HCU with one minor exception, in which SI was associated with fewer planned outpatient treatments (IRR = .97, 95% CI [.94, .99]).

**Conclusions:**

Our findings suggest that loneliness is a risk factor for certain types of HCU, independent of social isolation, baseline demographics, and chronic disease.

**Key messages:**

- The findings suggest that lonely individuals are slightly overrepresented within the healthcare system. In contrast, social isolation does not affect the use of health care services.
- The association between loneliness and health care utilization is complex and there is a need for future studies to investigate the mechanisms underlying the association.

